# Healthcare Professionals’ Knowledge, Attitudes and Counselling Practice Regarding Prevention of Secondhand Smoke Exposure Among Pregnant Women/Children in Assiut, Egypt

**DOI:** 10.3389/ijph.2022.1605073

**Published:** 2022-10-31

**Authors:** Zeinab M. Hassanein, Rachael L. Murray, Ilze Bogdanovica, Tessa Langley

**Affiliations:** ^1^ Public Health Department, Faculty of Medicine, Assiut University, Assiut, Egypt; ^2^ School of Medicine, Clinical Sciences Building, City Hospital, University of Nottingham, Nottingham, United Kingdom; ^3^ SPECTRUM Consortium, Clinical Sciences Building, City Hospital, University of Nottingham, Nottingham, United Kingdom

**Keywords:** pregnancy, children, Egypt, second-hand smoke, health care professionals

## Abstract

**Objectives and Methods:** A cross sectional study of HCPs working in public MCH clinics in Assiut city was conducted to explore their knowledge, attitudes and counselling practices regarding prevention of SHS exposure among pregnant women and children. Descriptive and regression analyses were performed.

**Results:** 367 HCPs participated in the study, 12% of whom were smokers. The majority were nurses (45%). A considerable proportion of HCPs reported being exposed to SHS in workplace (70%) and home (52%). About half HCP reported high SHS knowledge (56%), supportive attitude towards preventing SHS exposure (53%), and having good counselling practice regarding SHS exposure (52%). Being a GP and serving urban communities were significantly associated with high knowledge. Being female, serving a rural population, receiving training on smoking cessation services, not being exposed to SHS at home, and having a supportive attitude towards prevention of SHS exposure were significantly associated with good counselling practice.

**Conclusion:** Awareness, attitudes and counselling practice of HCPs should be improved. Training for HCPs and enforcement of smoke free polices are needed to improve awareness and facilitate changes in social norms.

## Introduction

Secondhand smoke (SHS) causes significant harm to children and adverse pregnancy outcomes [1–3]. The health care costs associated with treating health conditions due to SHS exposure are estimated to be approximately $7.1 billion in some Middle Eastern countries [4]. In Egypt, tobacco smoking is a widely accepted in homes and public places [5]. In 2018, 43.4% of males and 0.5% of females were smokers [6]. Despite, the presence of smoke-free legislations in Egypt and previous evidence linked between reductions in acute coronary event hospitalizations and implementation of smoke free legislation [7], in 2017 smoking was responsible for about 11% of disability adjusted life years (DALYs) and 17% of deaths in Egypt, and exposure to SHS was responsible for 16,000 deaths and 700,000 DALYs [8]. Low prevalence of smoking among females in Egypt is due to traditional gender roles which depict women’s smoking as disrespectful to society, and as a result there is stigma around women who smoke [9]. While only a small proportion of women are active smokers, the high rates of male smoking put non-smoking females at risk of SHS exposure.

In Egypt smoking is banned in indoor workplaces, public transport and indoor public places; however, there are no mechanisms or infrastructure to ensure enforcement of smoke-free legislation, and exposure to SHS is therefore high [10]. The prevalence of SHS exposure among women in reproductive age (15–49 years) in Egypt is estimated to be 65% [11], and about 50% of pregnant non-smoking women in Egypt are exposed daily to SHS [12] compared to 29% of non-smoking adults in European Union [13]. Pervious evidence reported significant relation between SHS exposure reductions on public places as school and in private places as cars and lower hospital admissions due to respiratory illness among children, following a comprehensive smoke-free policy [14]. In 2014, 35% of Egyptian school students (aged 13–15) were exposed to SHS at home and 55% in enclosed public places [15].

A lack of knowledge about the health risks of SHS for family members, especially children, is an important risk factor for SHS exposure [16–19]. Health care professionals (HCPs), especially nurses and midwives, are well placed to help reduce exposure to SHS in pregnant women and children. They spend a significant amount of time in contact with pregnant women and can therefore ask about their SHS exposure, advise them to prevent SHS exposure and encourage their husbands to quit smoking; this HCPs advice has been shown to be effective in previous studies [20–22].

This study aimed to explore the knowledge, attitudes and counselling practices of HCPs in maternal and child health (MCH) clinics in Egypt in relation to prevention of SHS exposure among pregnant women and children, and to identify the factors related to of their knowledge, supportive attitudes and counselling practices. We also aimed to explore barriers to the provision of counselling and the needs of HCPs in relation to improving the delivery of counselling on how to avoid SHS exposure to pregnant women and children.

## Methods

A cross-sectional survey of HCPs was undertaken in Assiut city, one of the largest cities in South Egypt. An anonymous self-administered paper-based questionnaire was distributed to all 535 HCPs working in all public MCH clinics in primary or secondary health care centres in Assiut city in August 2020. The study was approved by the School of Medicine and Health Sciences Research Ethics Committee at the University of Nottingham, United Kingdom and the Research Ethics Committee in the School of Medicine at Assiut University, Egypt.

### Instrument and Data Collection

A questionnaire development was guided by studies with similar research questions [23–28]. However, we did not perform a full validation procedure for the questionnaire; it was translated to Arabic by the lead researcher (ZH) then translated back into English by a second translator. It was also piloted on 15 HCPs in Egypt to determine the clarity of questions and length of time needed for questionnaire completion. The questionnaire collected data on socio-demographic and professional characteristics of HCPs, knowledge of HCPs regarding SHS exposure among pregnant women and children, barriers to the provision of counselling and perceived needs of HCPs to allow them to improve the delivery of counselling service to pregnant women and mothers to avoid SHS exposure.

### Data Management and Analysis

All data were entered into Microsoft Excel and then exported to STATA v.16 software for data management and analysis. Frequency distributions were used to summarise all variables. Similar to the approach used in previous studies [29–32], indices were created to summarise awareness, attitudes and practices. To summarise knowledge of HCPs, a knowledge index was constructed by adding the scores of individual items. A similar approach was used to create an attitude index. A high score on attitudes corresponded to a high level pronouncing a supportive attitude towards the prevention of smoking and SHS exposure among pregnant women and children. Similarly, a high score on practice corresponded to a high level of offering help (always or sometimes) to pregnant women/children to prevent SHS exposure among them by explaining the hazards of SHS and advising on how to avoid it. After creating scores for the three outcome measures (knowledge, supportive attitude towards prevention of SHS exposure, and counselling practice), each score was grouped into two categories, “high,” and “low” using the median of every score. As DeCoster, Iselin, and Gallucci (2009) [33] argued that dichotomization *via* the median split procedure or other cut-off points “makes analyses easier to conduct and interpret” especially if the underlying variable is naturally categorical, we used the median as a cut-off point to denote a “high” score for every index. The median of knowledge index was 9/12. The median for supportive attitude to prevention of SHS index was 7/10, and the median for counselling practice index was 3/5. The scores on the outcome measures were analysed separately (Supplementary Material S1).

Univariate logistic regression analysis was used to explore factors associated with high knowledge, supportive attitude and good counselling practice of HCPs on SHS exposure. The following variables were analysed: gender, age, specialty, smoking status, SHS exposure at workplace, SHS exposure at home, location of current medical practice i.e., urban/rural, years of post-graduate experience, and receiving previous training on smoking cessation service. Those variables that were statistically significant in univariate analysis at the *p* < 0.05 level were included in the multivariable logistic regression models using stepwise (forward) multivariable analyses to ascertain the factors associated with the three outcome variables (high knowledge, supportive attitude towards prevention of SHS exposure, and counselling practice). Odds ratios, 95% CI, and likelihood ratio test *p*-values for categorical exposure variables were reported. In the multivariable logistic regression model exploring good counselling practices, in addition to the variables included in univariate regression level, knowledge and supportive attitudes variables were included in the model as co-variates to explore the effect of knowledge and attitudes of HCPs on their counselling practice.

## Results

### Participant Demographics, Smoking Behaviours and Secondhand Smoke Exposure

Out of the 535 HCPs, 367 participated in the study with a response rate of 68.5% (Table 1). 44.7% were nurses, 20.4% were gynaecologists/obstetricians and 16.1% were paediatricians. A third were male and two-thirds served urban communities. 22% of study participants reported having received training on smoking cessation, mainly in the workplace. 12.5% of HCPs reported being smokers, 70.3% of study participants were exposed to SHS in their workplace and 51.8% in their homes (Table 1).

**TABLE 1:** Demographics, smoking behaviours, and SHS exposure of Health care professionals (Assiut, Egypt. 2022).

Demographic characteristics	N = 367	%
Specialty		
Gyn/obs	75	20.4
Paediatrician	59	16.1
GP	34	9.3
Nurse	164	44.7
Midwife	31	8.5
Other	4	1
Age		
<30	124	33.8
31–40	149	40.6
41–50	67	18.3
51–60	23	6.3
>60	4	1
Gender		
Male	118	32.1
Female	249	67.2
Current medical practice		
Rural	124	33.8
Urban	243	66.2
Post-graduate experience		
<5 years	100	27.3
5–10 years	109	29.7
>10 years	158	43
Previous training on smoking cessation service		
Yes	81	22.1
No	286	77.9
Type of training (N = 81 who responded yes to above question)		
During medical school	10	12.3
Post graduate clinical training	11	13.6
Training at work place	60	74.1
Smoking status		
Current smokers	46	12.5
Ex-smoker	9	2.5
Never smoker	312	85
Smoking in workplace (Total = 46 smoker)		
Yes	20	43.5
No	21	45.6
Prefer not to say	5	10.9
Intentions to quit smoking (Total = 46)		
I REALLY want to stop smoking and intend to in the next month	9	19.6
I REALLY want to stop smoking and intend to in the next 3 months	10	21.7
I want to stop smoking and hope to soon	12	26.1
I REALLY want to stop smoking but I do not know when I will	8	17.4
I want to stop smoking but have not thought about when	0	0
I think I should stop smoking but do not really want to	3	6.6
I do not want to stop smoking	2	4.3
I do not know	2	4.3
Exposure to SHS in your workplace		
Yes	258	70.3
No	109	29.7
Exposure to SHS in your home		
Yes	190	51.8
No	177	48.2

### Knowledge of Health Care Professionals Regarding Secondhand Smoke

Most of HCPs knew that SHS exposure increases the risk of congenital anomalies (78.7), low birth weight (76.8), spontaneous abortion (70.5), preterm delivery (69.9), sudden unexpected death in infancy (64.6), and death in infancy (64.6), and stillbirth in pregnant women (63.8). They knew that SHS increases the risk of respiratory tract infection (88.6), wheeze and asthma (80.4), chances of smoking uptake in the future(75.5%), and behavioural problems among children (68.1) (Table 2). A lower proportion were aware that SHS exposure among children increases the risk of middle ear infection (53.1%) and invasive meningococcal disease (28.6%).

**TABLE 2:** Health care professionals’ knowledge, attitude and practice regarding SHS exposure during pregnancy and childhood (Assiut, Egypt. 2022).

Health care professionals’ knowledge								
As far as you are aware, does SHS exposure during pregnancy increase the risk of the following?^a^			**Yes**		**No**		**Don’t Know**	
			**N**	**%**	**N**	**%**	**N**	**%**
Congenital anomalies			288	78.7	23	6.3	55	15
Low birth weight			282	76.8	9	2.5	76	20.7
Spontaneous abortion			258	70.5	31	8.5	77	21
Preterm delivery			256	69.9	30	8.2	80	21.9
Sudden unexpected death in infancy			237	64.6	26	7.1	104	28.3
Stillbirth			234	63.8	39	10.6	94	25.6
As far as you are aware, does SHS exposure among children increase the risk of the following?								
Respiratory tract infection			325	88.6	7	1.9	35	9.5
Wheeze and asthma			295	80.4	17	4.6	55	14.9
Chances of smoking uptake among children in the future			277	75.5	10	2.7	80	21.8
Psychological and behavioural problem			250	68.1	24	6.5	93	25.3
Middle ear infection			195	53.1	48	13.1	124	33.8
Invasive meningococcal disease			105	28.6	98	26.7	164	44.7
**Health care professionals’ attitudes**								
To what extent do you agree with this statement?^a^			**Agree**		**Disagree**		**Unsure**	
			**N**	**%**	**N**	**%**	**N**	**%**
Health care professionals should not smoke as patients could see them as role models			339	92.4	11	3	17	4.6
Health professionals should routinely advise pregnant women/mothers with children to avoid SHS exposure			339	92.4	3	0.8	25	6.8
Health professionals should routinely ask pregnant women/mothers with children about whether they are exposed to SHS			330	89.9	6	1.6	31	8.5
Compared with other disease prevention activities like obesity and hypertension, tobacco control is important			330	89.9	7	1.9	30	8.2
A pregnant woman’s/child’s chances of avoiding SHS exposure could increase if a health professional advises pregnant women/mothers with children to avoid it			320	87.1	2	0.5	45	12.3
Health professionals who smoke are less likely to advise pregnant women/mothers with children to avoid SHS exposure			288	78.5	47	12.8	32	8.7
SHS exposure is private business, therefore there should be no advice from HCPs regarding this topic			128	34.9	215	58.6	24	6.5
Pregnant women/mothers with children are not interested in receiving advice about reducing SHS exposure			172	46.9	106	28.9	89	24.3
Giving advice on avoiding SHS exposure has a low chance of success			167	45.5	102	27.8	98	26.7
In the course of my profession there are other aspects more important than SHS exposure			199	54.2	99	26.9	69	18.8
**Health care professionals’ counselling practice**								
To what extent do you practice the following?^a^	**Always**		**Sometimes**		**Rarely**		**Never**	
	**N**	**%**	**N**	**%**	**N**	**%**	**N**	**%**
I ask pregnant women/mother with children if they are exposed to SHS.	32	8.7	139	37.9	80	21.8	116	31.6
I explain the consequences of SHS on one’s health to pregnant women/mother with children	60	16.4	136	37.1	132	35.9	39	10.6
I explain the specific adverse health effects of SHS exposure to the foetus during pregnancy	75	20.4	121	32.9	98	26.7	73	19.9
I explain the specific adverse health effects of children’s SHS exposure to their mothers	68	18.5	107	29.2	103	28.1	89	24.3
I advise/encourage pregnant women/mother with children to avoid SHS exposure	85	23.2	129	35.2	69	18.8	84	22.9

^a^ Total N = 367.

55.9% of study participants had high knowledge of the dangers of SHS exposure to health of pregnant women and children. Being a General Practitioner (GP) (OR 15.29, 95%CI 4.12–56.86), serving urban communities (OR 2.53, 95%CI 1.53–4.18) and being exposed to SHS at home (OR 2.36, 95%CI 1.48–3.78) were significantly associated with high knowledge (Table 3). The strongest observed association was for GPs who were more than 15 folds compared to obstetricians and gynaecologists (95%CI 4.12–56.86) to have high knowledge after adjustment for current medical practice and SHS exposure at home.

**TABLE 3:** Multivariable regression of factors associated with knowledge, attitude and counselling practice of HCPs regarding prevention of SHS exposure among pregnant women and children (Assiut, Egypt. 2022).

	Total	Good knowledge					Good supportive attitude					Good counselling practice				
			Univariate analysis		Multivariable model^a^			Univariate analysis		Multivariable model^b^			Univariate analysis		Multivariable model^c^	
	N 367	N (%) 205 (55.86)	OR	95%CI	Adjusted OR	95%CI	N (%) 194 (52.9)	OR	95%CI	Adjusted OR	95%CI	N (%) 190 (51.8)	OR	95%CI	Adjusted OR	95% CI
Gender																
Male	118	79 (66.9)	1.98*	1.25–3.12			51 (43.2)	1.00		1.00		50 (42.4)	1.00		1.00	
Female	249	126 (50.6)	1.00				143 (57.4)	1.77*	1.14–2.74	2.02	1.27–3.24	140 (56.2)	1.75*	1.12–2.72	1.53	1.15–2.63
Age																
<30	124	84 (67.7)	1.00				67 (54)	1.00				58 (46.8)	1.00			
31–40	149	72 (48.3)	0.45*	0.27–0.73			84 (56.4)	1.11	0.68–1.77			86 (57.7)	1.55	0.96–2.5		
41–50	67	36 (53.7)	0.55*	0.30–1.02			30 (44.8)	0.69	0.38–1.25			35 (52.3)	1.2	0.89–2.25		
51–60	23	10 (43.5)	0.37*	0.15–0.91			12 (52.2)	0.93	0.38–2.26			10 (43.5)	0.88	0.36–2.15		
>60	4	3 (75)	1.43*	0.14–14.17			1 (25)	0.28	0.03–2.80			1 (25)	0.38	0.04–3.75		
Specialty																
Gyn/obs	75	36 (48)	1.00		1.00		42 (56)	1.00				41 (54.7)	1.00			
Paediatrician	59	43 (72.9)	2.9*	1.36–6.21	3.15	1.48**–**6.72	26 (44.1)	0.62	0.31–1.23			30 (50.9)	0.86	0.43–1.69		
GP	34	31 (91.2)	11.19*	2.77–45.31	15.29	4.12**–**56.86	20 (58.8)	1.12	0.49–2.55			14 (41.2)	0.58	0.26–1.32		
Nurse	164	80 (48.8)	1.03*	0.60–1.8	1.09	0.60**–**1.99	85 (51.8)	0.85	0.48–1.46			85 (51.9)	0.89	0.52–1.45		
Midwife	31	14 (45.2)	0.89*	0.38–2.1	1.12	0.45**–**2.79	20 (64.5)	1.43	0.60–3.39			18 (58.1)	1.15	0.49–2.68		
Others	4	1 (25)	0.36*	0.034–3.73	0.35	0.3**–**3.66	1 (25)	0.26	0.02–2.63			2 (50)	0.83	0.11–6.2		
Current medical practice																
Rural	124	52 (41.9)	1.00		1.00		76 (61.3)	1.68*	1.08–2.6	1.59	1.01–2.49	85 (68.6)	2.86*	1.82–4.52	2.32	1.37–3.94
Urban	243	153 (62.9)	2.35*	1.51–3.69	2.53	1.53–4.18	118 (48.6)	1.00		1.00		105 (43.2)	1.00		1.00	
Post-graduate experience																
<5 years	100	67 (67)	1.00				54 (54)	1.00				47 (47)	1.00			
5–10 years	109	72 (66)	2.71*	1.65–4.86			47 (43.1)	0.65*	0.37–1.11			51 (46.8)	0.99	0.58–1.7		
>10 years	158	66 (41.7)	2.83*	1.61–4.58			93 (58.9)	1.22*	0.74–2.02			92 (58.2)	1.57	0.95–2.6		
Previous training on smoking cessation service																
Yes	81	42 (51.9)	1.00				49 (60.5)	1.00				59 (72.8)	3.17*	1.85–5.46	2.79	1.5–5.21
No	286	163 (56.9)	1.23	0.75–2.02			145 (50.7)	0.67	0.41–1.1			131 (45.8)	1.00		1.00	
Smoking status																
Never smoker	312	168 (53.9)	1.00				168 (53.9)	1.00				161 (51.6)	1.00			
Ex-smoker	9	8 (88.9)	6.86*	0.85–55.48			4 (44.5)	0.69	0.18–2.6			4 (44.4)	0.75	0.18–2.84		
Current smoker	46	29 (63)	1.46*	0.77–2.77			22 (47.9)	0.79	0.42–1.46			25 (54.4)	1.12	0.59–2.08		
SHS exposure at workplace																
No	109	49 (44.9)	1.00				64 (58.7)	1.00				56 (51.4)	1.00			
Yes	258	156 (60.5)	1.87*	1.19–2.96			130 (50.4)	0.71	0.45–1.2			134 (51.9)	1.02	0.65–1.6		
SHS exposure at home																
No	177	83 (46.9)	1.00		1.00		106 (59.9)	1.73*	1.14–2.62	2.36	1.29–3.10	109 (61.6)	1.16*	1.42–3.28	2.29	1.37–3.83
Yes	190	122 (64.2)	2.03*	1.33–3.11	2.36	1.48–3.78	88 (46.3)	1.00		1.00		81 (42.6)	1.00		1.00	
Knowledge																
Inadequate	162											97 (59.8)	1.00			
Good	205											93 (45.4)	0.95	0.53–1.68		
Supportive attitude																
Inadequate	173											51 (29.5)	1.00		1.00	
Good	194											139 (71.7)	6.05*	3.85–9.50	5.49	3.38–8.90

Bold values or * are when *p* value of likelihood ratio test is significant; *p* value ≤0.05.

^a^ Multivariable model adjusted for speciality, current medical practice, and SHS exposure at home.

^b^ Multivariable model adjusted for gender, current medical practice, and SHS exposure at home.

^c^ Multivariable model adjusted for gender, current medical practice, previous training on smoking cessation service, SHS exposure at home, knowledge, and supportive attitude.

### Attitudes of Health Care Professionals Towards Smoking and Secondhand Smoke Exposure Among Pregnant Women and Children

34.9% of HCPs agreed that SHS exposure is private business and 45.5% agreed that giving advice on avoiding SHS exposure has a low chance of success (Table 2), reflecting the limited supportive attitude of HCPs towards prevention of SHS exposure among pregnant women and children.

Only 52.9% of HCPs had a supportive attitude towards the prevention of smoking and SHS exposure among pregnant women or children. Being female (OR 2.02, 95%CI 1.27–3.24), serving rural communities (OR 1.59, 95%CI 1.01–2.49), and not being exposed to SHS at home (OR 2.36, 95%CI 1.29–3.10) were significantly associated with a supportive attitude (Table 3). The strongest observed association was for those not exposed to SHS at home who were more than two folds compared to those exposed to SHS at home (OR 2.36, 95%CI 1.29–3.10) to have supportive attitude towards prevention of smoking and SHS exposure among pregnant women and children.

### Counselling Practice of Health Care Professionals Regarding Prevention of Secondhand Smoke Exposure Among Pregnant Women and Children

About half of HCPs mentioned that they sometimes or always ask pregnant women/mothers with children if they are exposed to SHS (46.6%), explain the consequences of SHS on health (53.4%), explain the specific adverse health effects of SHS exposure to the foetus during pregnancy (53.4%), explain the specific adverse health effects of SHS on health of children (47.7%), and advise/encourage pregnant women/mother with children to avoid SHS exposure (58%) (Table 2).

About half of HCPs (51.8%) reported good counselling practice regarding counselling pregnant women/mothers with children about SHS exposure (Table 3). Being female (OR 1.88, 95%CI 1.15–3.07), serving a rural population (OR 2.44, 95%CI 1.51–3.96), receiving previous training on smoking cessation services (OR 2.59, 95%CI 1.45–4.61), not being exposed to SHS at home (OR 2.66, 95%CI 1.68–4.22), and having a supportive attitude (OR 5.49, 95%CI 3.38–8.90) towards prevention of SHS exposure were significantly associated with good counselling practice. The strongest observed association was for those having a supportive attitude towards the prevention of SHS exposure, who after adjusting for covariates were more than five folds compared to those do not have supportive attitude towards the prevention of SHS exposure (OR 5.49, 95%CI 3.38–8.90) to have good counselling practice.

### Barriers to Provision of Counselling and Needs of Health Care Professionals to Improve the Delivery of Counselling

Lack of time or training, absence of reimbursement and unavailability of materials were the most common barriers to the provision of counselling (Table 4). Lack of time was the first barrier for most of gynaecologists/obstetricians (57.3%), paediatricians (72.9%), and GPs (67.7%). However, lack of training was the first barrier for most nurses (64%) and midwives (54.8%). The majority of HCPs (75%) suggested that it is nurses’ job to discuss SHS exposure with pregnant women/mothers with children. The majority of participants stated that they need training, standard guidelines and materials about SHS health hazards to help them improve the delivery of counselling on SHS. HCPs reported that health education sessions for pregnant women/mothers of children and smokers in their household could help them to reduce SHS exposure.

**TABLE 4:** Barriers to provision of counselling and needs of HCPs to improve the delivery of counselling service (Assiut, Egypt. 2022).

Barriers for HCPs to advise pregnant women/mothers with children to avoid SHS exposure	N = 367^a^	%
Lack of time	228	62.1
Lack of training	195	53.1
There is no reimbursement for advising women to avoid SHS exposure	167	45.5
Unavailability of materials (e.g., brochures about health hazards of SHS)	147	40.1
Low chances of success	122	33.2
Pregnant women/mothers with children do not want/expect to receive that advice	92	25.1
SHS exposure counselling is not a part of my job	69	18.8
Feeling uncomfortable discussing as I think it is a sensitive topic	57	15.5
**HCPs’ opinion regarding barriers for pregnant women/mothers with children to avoid SHS exposure**		
Husband smoking at home	317	86.4
Ignorance of the risks of SHS exposure	274	74.7
Another household smoker	221	60.2
Lack of self-confidence to ask smoker in her household to stop smoking	187	50.9
Smoking being accepted in the society	186	50.7
Regulations on smoking in public places are not enforced	181	49.3
Societal attitudes towards women asking her husband/smoker in her household to stop smoking	116	31.6
Other	1	0.3
**Whose job is it to discuss SHS exposure with pregnant women/mothers with children**		
Nurse	276	75.2
Midwife	200	54.5
General practitioner (GP)	184	50.1
Others	53	14.4
**What do HCPs’ need to deliver/improve the delivery of SHS counselling service among pregnant women/mothers of children?**		
Training for HCPs	307	83.7
Availability of standard guidelines in the health centre	237	64.6
Availability of materials about SHS health hazards	211	57.5
Nothing	7	1.9
Other	4	1.3
**What is the best way to help pregnant women/mothers with children to avoid SHS exposure?**		
Health education sessions for pregnant women/mothers of children	254	69.2
Health information materials for pregnant women/mothers of children	256	69.8
Health education sessions for pregnant women and their household smokers	210	57.2
Offering counselling sessions and nicotine replacement therapy to household smokers	181	49.3
Other	7	1.9

^a^ Respondents were allowed to choose many options.

## Discussion

The main findings of this study are that only about half of HCPs in Assiut city in Egypt have good risk awareness (55.9%), a supportive attitude (52.9%), and report good counselling practice (51.8%) regarding the prevention of SHS exposure among pregnant women and children. GPs and paediatricians were found to be most aware of the risks of SHS. Female HCPs were more likely to report good counselling practice. HCPs serving a rural population were most likely to have a supportive attitude for the prevention of SHS and report good counselling practice. HCPs who are not exposed to SHS at home were more likely to report good counselling practice and supportive attitude for its prevention among pregnant women and children.

Our results are consistent with other studies in Egypt and neighbouring countries which have reported that HCPs have vague or inaccurate knowledge about the risk of SHS and poor counselling practice in relation to SHS exposure [34–36]. Previous studies in Egypt reported better knowledge of the dangers of smoking and more supportive attitudes in relation to the provision of smoking cessation services among HCPs [23, 37]; however, those studies did not investigate in details the knowledge regarding the specific dangers of SHS to pregnant women and children which highlight the novelty of our study. This difference could be due to these existing studies being not specific to SHS and being performed in one university hospital and urban family medicine centers in Alexandria, as opposed to a combination of urban and rural clinics as in the present study, in which HCPs serving rural communities showed lower knowledge.

The limited awareness of the health risks of SHS may be partly due to a lack of relevant training. Only one in five participants in the current study had previous training on smoking cessation, whether during medical school, post graduate clinical training or training at the workplace and receiving this training was significantly was significantly associated with good counselling practice of HCPs with pregnant women and children regarding their SHS exposure. This figure is lower than previously reported [23, 37] possibly due to the limited training programs on smoking cessation in South Egypt governorates. In the current study, lack of training was the first barrier for most nurses and midwives to provide the SHS counselling service suggesting that improvement in training provided to nurses could help to reduce SHs exposure.

It is important to ensure that the wider environment is conducive to increased awareness and willingness to provide support on smoking cessation and prevention of SHS exposure. This includes proper enforcement of smoke-free policy enshrined in law, and other population-level interventions such as mass media campaigns to make the social norms against SHS exposure. In combination with additional training, this can improve the knowledge and attitudes of HCPs, as well as the general population, and change counselling practice of HCPs.

Although Egypt has made important strides in controlling tobacco use according to World Health Organization’s Framework Convention on Tobacco Control (WHO FCTC) report [6], SHS exposure remains extremely high (more than 70%) in public places such as restaurants, public transportation, and health care facilities [5, 38] as the smoke-free legislation is poorly enforced [10]. This is comparable with our results as 70% of HCPs reported exposure to SHS in the workplace. While efforts to support the provision of advice related to SHS is likely to help reduce SHS exposure, these are likely to be most effective if they are made in the context of effective implementation of tobacco control policies, particularly the enforcement of smoke-free legislation.

In the current study about half of HCPs agreed that giving advice on avoiding SHS exposure is unlikely to be successful; this could be because HCPs claimed that they do not have time, training, and materials to deliver this service, or due to a lack of understanding of the effect that such advice may have. One third disagreed that pregnant women/mothers with children are interested in receiving advice about reducing SHS exposure. As evidenced from previous systematic review, smoking and SHS exposure is socially accepted in many Middle Eastern countries [39]. Therefore, proper enforcement of smoke-free law is expected to contribute to changes in social norms which will facilitate changes in SHS exposure. Enforcement of smoke-free policy could make women more interested in avoiding SHS exposure and could make HCPs feel offering advice can be helpful. Thus, overall environment is conducive to HCPs giving this sort of advice.

In the present study, the main obstacles for HCPs to help pregnant women/children to avoid SHS exposure were found to be lack of time, lack of training, absence of reimbursement and unavailability of materials. Similar obstacles have been reported in other middle income countries [40]. Previous evidence suggest that providing training for HCPs encourage them to provide counselling service to pregnant women to adopt smoke-free environment [41]. Training of HCPs cannot work alone. A range of issues need to be addressed including lack of time and unavailability of materials. Additionally, ensuring that HCPs in Egypt have the time and financial resources needed to deliver this type of support is essential. Clear specification of SHS counselling service in the job description of HCPs working in public MCH clinics should be performed by the health system governors. In this study, the majority of HCPs suggested that it is nurses’ job to discuss SHS exposure with pregnant women, so there is no clear description on whose job it is to do counselling service. However, previous evidence reported that nurses and physicians are ideally placed to provide health advice to pregnant women and mothers with children to influence their SHS exposure [42]. Thus, all HCPs in public MCH clinics need training to address their view that it is solely nurses’ responsibility to discuss SHS exposure and encourage them to discuss SHS exposure with their patients.

Previous studies have shown that pregnant women who do not smoke are often responsive to counselling regarding reduction of SHS exposure received from HCPs in antenatal care clinics [43, 44]. Moreover, studies have reported that counselling pregnant women not only led to reduction in their SHS exposure but also increased smoking cessation among their husbands, as well as increasing positive attitudes and practices to reduce SHS at home [43, 45]. Support from HCPs may therefore contribute to the reduction of SHS exposure in Egypt.

### Strengths and Limitations

To our knowledge, this is the first study that provides detailed evidence on the knowledge, attitudes and practice of Egyptian HCPs regarding SHS exposure among pregnant women and children. This study achieved a high response rate by distributing paper questionnaires, though this meant that the study focussed on HCPs working in only one governorate. Despite this, the study included both urban and rural areas. Furthermore, Assiut is the largest city in Upper Egypt, however the results may not be generalizable because of differences in sociodemographic characteristics between Assiut and other cities in Egypt. A further limitation, particularly in relation to assessing counselling practice of HCPs, is that the study findings are based on self-report. However, the study identified clear shortcomings in counselling practice, which are unsurprising given the low levels of knowledge and supportive attitudes to SHS prevention. Another limitation of bias that could be due to that the study main respondents were non-smokers and females, however, we performed multivariable regression analysis and models were adjusted for main demographic characteristics. Although, dichotomizing a variable based on cut-offs can jeopardize model fit and lead to misleading interpretation of results, we have performed a sensitivity analysis to ensure that the median cut-off point used in this study is not leading to misinterpretations (Supplementary Material S2).

### Conclusion and Recommendation

Awareness, attitudes and counselling practice of HCPs regarding the risks of SHS to pregnant women and children in Egypt should be improved. It is important to develop an environment which facilitates increased awareness of and willingness to provide support on smoking cessation and prevention of SHS exposure. This includes comprehensive enforcement of smoke-free policy and training programs for HCPs on smoking cessation which should cover SHS exposure. This could also extend to other population-level interventions such as mass media campaigns. Other barriers, such as the lack of time must also be addressed. More qualitative studies in Egypt are needed also to explore women’s views and experiences regarding their SHS exposure and the barriers to preventing this exposure among pregnant women and children.
