# Supplementary material for: Healthcare Professionals’ Knowledge, Attitudes and Counselling Practice Regarding Prevention of Secondhand Smoke Exposure Among Pregnant Women/Children in Assiut, Egypt
Source: Int J Public Health. 2022 Oct 31;67:1605073. doi: 10.3389/ijph.2022.1605073 (PMC9661921; doi:10.3389/ijph.2022.1605073)
Supplement: Supplementary file 1 [file DataSheet2.pdf]

Online supplement 2: Sensitivity analysis of knowledge, attitude, and counselling practice regression models of HCPs (Assiut, Egypt. 2022)

Sensitivity analysis of knowledge level multivariable regression model:

Classified	True		Total
	D	~D	
+	152	52	204
-	53	110	163
Total	205	162	367

Classified + if predicted Pr (D) >= 0.5

True D defined as Knowledge level! = 0

Sensitivity	Pr(+| D)	74.15%
Specificity	Pr(-|~D)	67.90%
Positive predictive value	Pr(D| +)	74.51%
Negative predictive value	Pr(~D| -)	67.48%
Correctly classified		71.39%

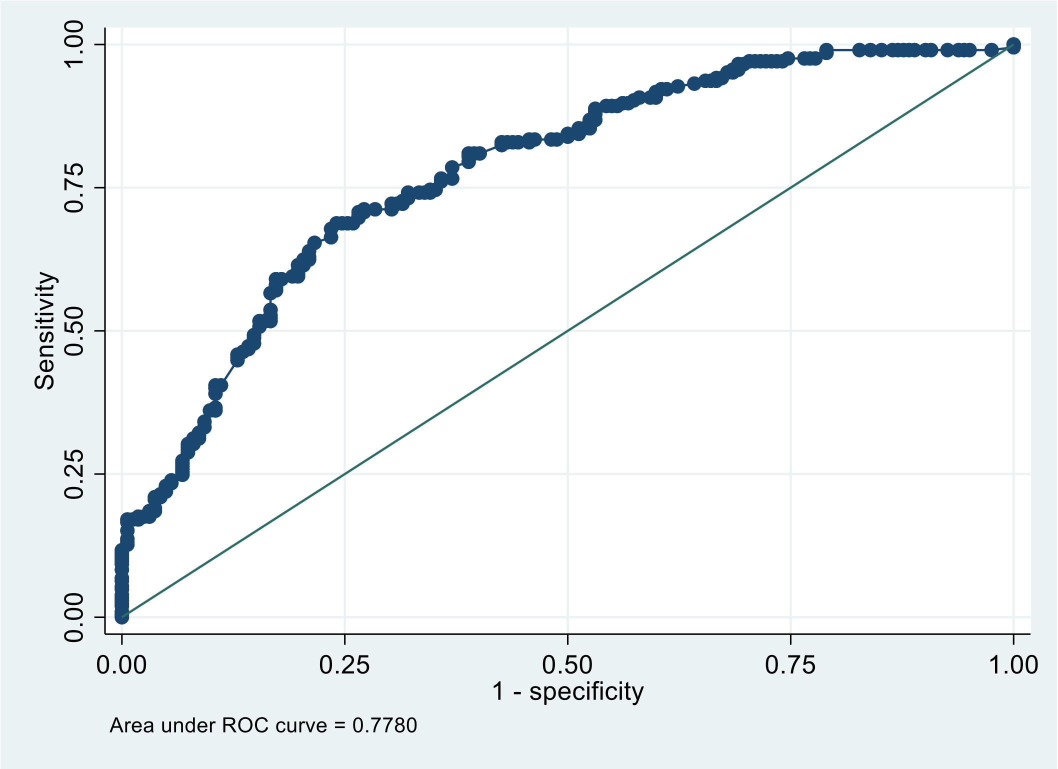

Sensitivity analysis of attitude level multivariable regression model:

Classified	True		Total
	D	~D	
+	133	79	212
-	61	94	155
Total	194	173	367

Classified + if predicted Pr (D) >= 0.5

True D defined as Knowledge level! = 0

Sensitivity	Pr(+| D)	68.56%
Specificity	Pr(-|~D)	54.34%
Positive predictive value	Pr(D| +)	62.74%
Negative predictive value	Pr(~D| -)	60.65%
Correctly classified		61.85%

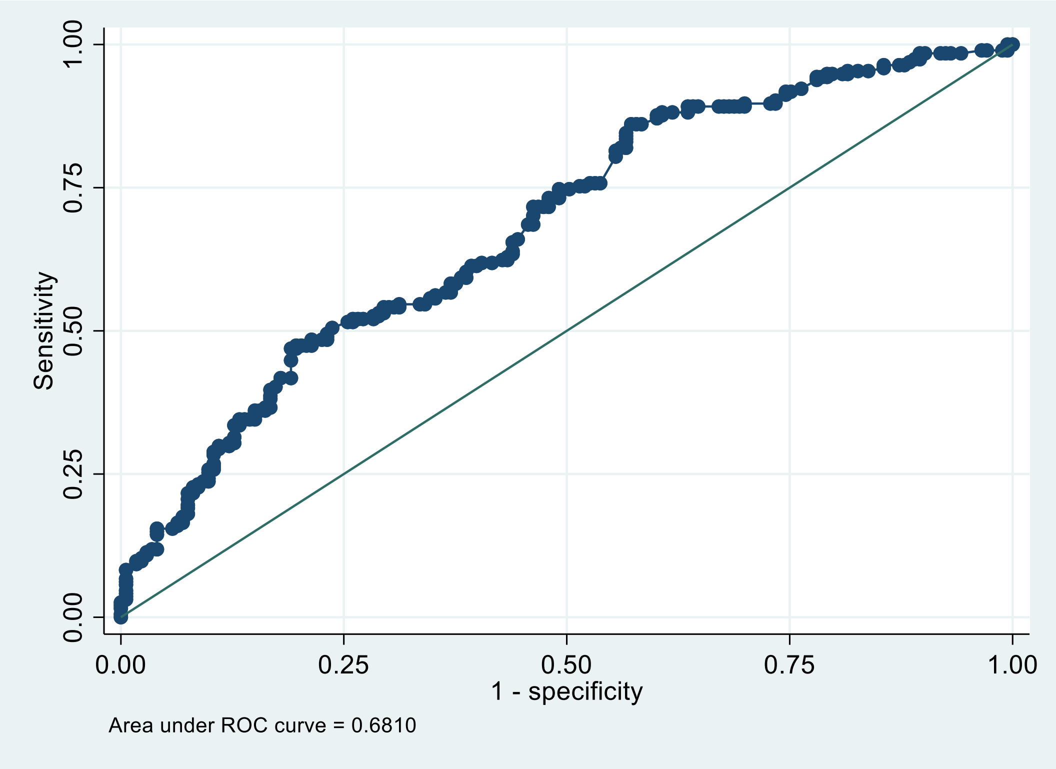

Sensitivity analysis of counselling practice level multivariable regression model:

Classified	True		Total
	D	~D	
+	146	50	196
-	44	127	171
Total	190	177	367

Classified + if predicted Pr (D) >= 0.5

True D defined as Knowledge level! = 0

Sensitivity	Pr(+| D)	76.84%
Specificity	Pr(-|~D)	71.75%
Positive predictive value	Pr(D| +)	74.49%
Negative predictive value	Pr(~D| -)	74.27%
Correctly classified		74.39%

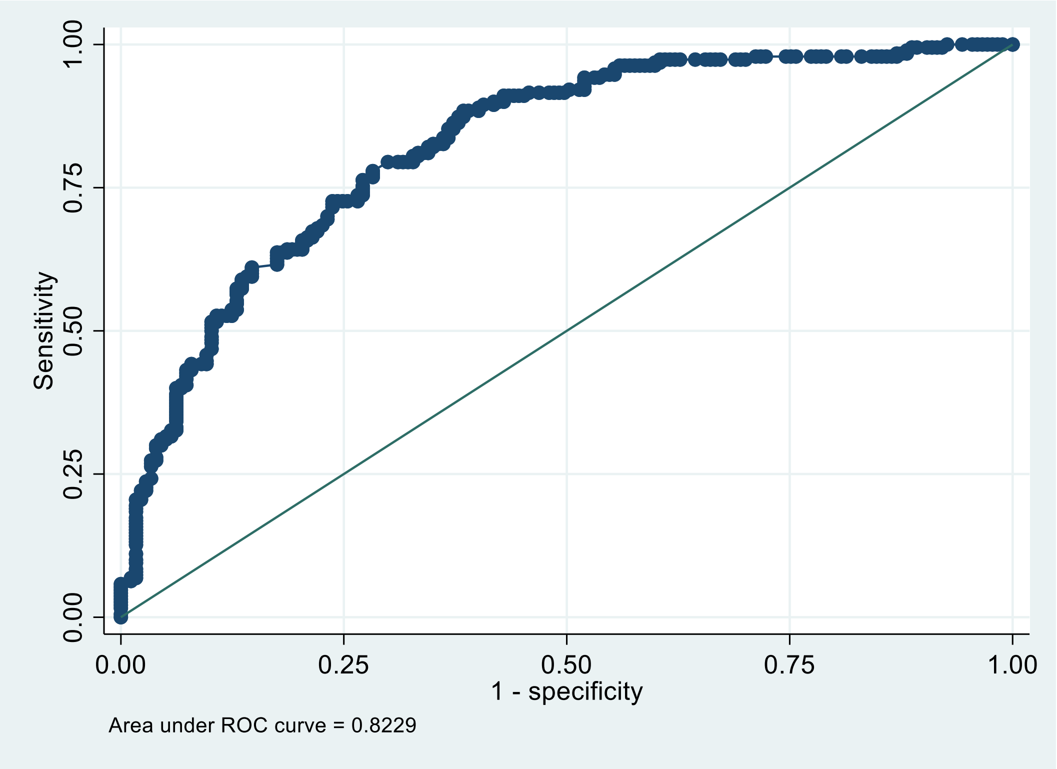

Based on a rough classifying system, Area under ROC curve (AUC) can be interpreted as follows: 90 -100 = excellent; 80 - 90 = good; 70 - 80 = fair; 60 - 70 = poor; 50 - 60 = fail.

So, knowledge and attitude regression analysis are considered acceptable. counselling practice regression analysis is considered good.
