# Supplementary material for: Healthcare Professionals’ Knowledge, Attitudes and Counselling Practice Regarding Prevention of Secondhand Smoke Exposure Among Pregnant Women/Children in Assiut, Egypt
Source: Int J Public Health. 2022 Oct 31;67:1605073. doi: 10.3389/ijph.2022.1605073 (PMC9661921; doi:10.3389/ijph.2022.1605073)
Supplement: Supplementary file 2 [file DataSheet1.pdf]

Online supplement 1: summary of knowledge, attitude and counselling practice indices of HCPs (Assiut, Egypt. 2022)

Online Table 1: Summary of Knowledge index of HCPs regarding SHS exposure among pregnant women/children (Assiut, Egypt. 2022)

Score	Frequency	%
Knowledge index (12 questions)		
0	8	2.18
1	7	1.91
2	12	3.27
3	22	5.99
4	24	6.54
5	23	6.27
6	20	5.45
7	18	4.90
8	28	7.63
9	31	8.45
10	41	11.17
11	61	16.62
12	72	19.62
Total	367	100
Median of knowledge index: 9		

Online Table 2: Summary of supportive attitude index of HCPs regarding prevention of SHS exposure among pregnant women/children (Assiut, Egypt. 2022)

Score	Frequency	%
Supportive attitude index (10 questions)		
0	4	1.09
1	1	0.27
2	4	1.09
3	10	2.72

4	12	3.27
5	26	7.08
6	116	31.61
7	70	19.07
8	72	19.62
9	31	8.45
10	21	5.72
Total	367	100
Median of supportive attitude regarding prevention of SHS index: 7		

Online Table 3: Summary of counselling practice index of HCPs regarding prevention of SHS exposure among pregnant women/children (Assiut, Egypt. 2022)

Score	Frequency	%
Counselling practice index (5 questions)		
0	134	36.51
1	15	4.09
2	28	7.63
3	20	5.45
4	29	7.90
5	141	38.42
Total	367	100
Median of Counselling practice regarding prevention of SHS index: 3		
